# Nutritional, Mineral, Taste, and Metabolic Profiles of *Agaricus bisporus* Cultivated on Corn Stover Substrate: A High-Quality Alternative to Wheat Straw

**DOI:** 10.3390/foods15142570

**Published:** 2026-07-22

**Authors:** Keqing Qian, Weijian Li, Dongyan Sun, Zhengxiang Qi, Peng Dong, Ze Liu, Ke Ma, Jingyu Wang, Yu Li, Han Yu, Xiao Li, Bo Zhang

**Affiliations:** 1 Sanjiang Laboratory, Jilin Agricultural University, Changchun 130118, China; 2 Engineering Research Center of Edible and Medicinal Fungi, Ministry of Education, Jilin Agricultural University, Changchun 130118, China; 3 College of Mycology, Jilin Agricultural University, Changchun 130118, China; 4 Industrial Development Institute for Plants, Animals and Fungi Integration of Biyang County, Biyang 463799, China; 5 College of Agriculture, Jilin Agricultural University, Changchun 130118, China; 6 Jilin Academy of Agricultural Sciences, Changchun 130033, China

**Keywords:** edible mushroom, stover, nutrition, electronic tongue, metabolomics

## Abstract

This study systematically evaluated substrate-dependent variations in yield, nutritional composition, mineral profiles, taste-active compounds, electronic tongue profile, and metabolome of *A. bisporus* cultivated on corn stover-based substrates (CM1, CM2, CM3) and wheat straw-based substrate (WM4). Corn stover-based substrates supported the cultivation of *A. bisporus*, yielding 20.56–25.63 kg/m^2^, comparable to WM4 (24.23 kg/m^2^). Biological efficiency of the corn stover treatments ranged from 80.32% to 88.58%; CM1 and CM2 were comparable to WM4 (91.35%), whereas CM3 showed a significantly lower biological efficiency than WM4 (*p* < 0.05). Crude protein and mineral elements (Cu, Fe, Zn) were substantially higher in mushrooms from corn stover-based substrates, whereas crude polysaccharide content was greater in WM4. Taste profile analysis revealed that corn stover-based substrates elevated umami and sweet amino acids alongside 5′-GMP and 5′-UMP, leading to enhanced equivalent umami concentration (EUC) and umami perception by the electronic tongue, while WM4 supported higher 5′-AMP accumulation. Non-targeted metabolomics analysis (CM3 vs. WM4) identified 3997 metabolic features. KEGG pathway enrichment analysis revealed that these substrate-dependent metabolic alterations were primarily driven by pathways such as the biosynthesis of amino acids, nucleotide metabolism, and 2-oxocarboxylic acid metabolism, underlying the metabolic adaptation of *A. bisporus*. These findings provide new insights into the compositional characteristics and quality formation of *A. bisporus* cultivated on corn stover-based substrates.

## 1. Introduction

A fivefold increase in global mushroom production was recorded during the 2000–2023 period, with output levels attaining 44 million tons [1]. Among these, white button mushrooms (*Agaricus bisporus*) represent one of the most extensively cultivated and consumed edible mushrooms worldwide [2,3]. *Agaricus bisporus* is rich in polysaccharides, minerals, dietary fiber, essential amino acids, polyphenols, and various micronutrients [4,5]. Its exceptional nutritional profile, low fat content, palatable texture, and distinctive umami flavor have garnered widespread consumer appeal globally [6,7]. As an economically significant crop and a valuable source of high-quality protein and bioactive compounds, *A. bisporus* has been demonstrated to possess immunomodulatory, antioxidant, and metabolic health benefits. Consequently, research aimed at optimizing its cultivation is essential to meet the increasing global demand while enhancing its nutritional and functional attributes [8,9].

The nutritional quality, mineral contents, and flavor characteristics of edible mushrooms are largely dependent on the composition and properties of their cultivation substrates [10]. These substrates not only provide essential nutrients for mycelial growth and fruiting body development but also regulate fungal metabolic pathways through complex molecular mechanisms, influencing nutrient accumulation and flavor compound formation [11]. The substrate-specific effects on mushroom quality have been extensively documented across multiple species. In cultivation experiments with oyster mushrooms and *Pleurotus cystidiosus*, replacing traditional 100% sawdust with either 100% sugarcane bagasse or 100% corn cob led to significant improvements in nutritional parameters, including higher protein, fiber, ash, and mineral content (calcium, potassium, magnesium) [12]. Similarly, comparative studies on *Pleurotus pulmonarius* grown on three forestry wastes revealed that the first-flush mushrooms that were grown on the pine sawdust-based substrate had the highest protein content. In contrast, those on the honeysuckle rattan sawdust-based substrate had the highest gamma-aminobutyric acid content and equivalent umami concentration values [13]. In *A. bisporus*, partial substitution of soybean meal with tea residue increased amino acid and key volatile flavor compound levels [14].

Substrate composition serves as a critical determinant of mushroom quality regulation, modulating nutritional value and flavor characteristics through the activation of specific metabolic pathways. Different substrates induce differential expression in carbon metabolism, amino acid biosynthesis, and secondary metabolism. Recent metabolomics investigations revealed 439 differentially abundant metabolites in mushroom fruiting bodies cultivated on different substrate types—Korshinsk peashrub (KP) versus conventional apple wood sawdust, alongside a 1.5-fold increase in protein and lipid content. Integrated metabolomics and transcriptomics analyses identified 698 upregulated and 498 downregulated genes associated with these metabolic alterations, indicating that reprogrammed carbon metabolism and oxidoreductase pathways play pivotal roles in mushroom responses to substrate variation [15].

The current *A. bisporus* industry continues to face significant challenges in substrate selection and nutritional quality optimization. Traditional cultivation practices predominantly rely on substrates such as wheat straw, which pose considerable limitations, including regional constraints, seasonal availability fluctuations, and uneven geographic distribution. Furthermore, substantial opportunities remain to enhance the nutritional composition and flavor profile optimization. Concurrent with China’s agricultural structural reforms and crop production development, alternative substrate resources such as corn stover have become increasingly abundant. Since 2008, corn has surpassed rice as the primary contributor to total crop residues, accounting for 38.9% of all collectible crop residues generated in 2020, while wheat production increased from 20.5% in 2001 to 22.8% in 2020 [16]. Statistical analyses across China’s six major agricultural regions demonstrate that corn stover exhibits a more uniform and extensive distribution pattern across these regions, encompassing territories from the northern Northeast and North China regions to the southern South China region. In contrast, wheat straw distribution remains predominantly concentrated in northern and central China, with markedly reduced availability in southern regions. Corn stover presents distinct advantages, including high yield potential and widespread geographic distribution [17]. Chemically, it is distinguished by a significantly higher content of nitrogen and water-soluble carbohydrates compared to wheat straw [18].

Despite significant advances in mushroom substrate optimization research, quantitative comparisons between corn stover and conventional wheat straw substrates in *A. bisporus* cultivation remain scarce, especially concerning mushroom quality enhancement. Furthermore, the substrate-specific mechanisms governing nutritional and taste profile development are largely unexplored. To the best of our knowledge, few studies have systematically compared corn stover and wheat straw substrates in terms of their integrated effects on proximate composition, mineral profiles, free amino acids, and umami compounds in *A. bisporus*. By integrating bacterial microbiome sequencing, metabolomics analysis, and electronic tongue taste analysis, the metabolic mechanisms underlying substrate-dependent variations were investigated.

## 2. Materials and Methods

### 2.1. Standards and Reagents

Element standard solutions (1000 µg/mL) were obtained from the National Nonferrous Metals and Electronic Materials Analysis and Testing Center (Beijing, China). Amino acid standards were supplied by Anpel Laboratory Technologies Inc. (Shanghai, China). HPLC-grade acetonitrile was obtained from Merck KGaA (Darmstadt, Germany). All other reagents were of at least analytical grade.

### 2.2. Cultivation of Agaricus bisporus

Corn stover was utilized as the primary carbon source for the experimental groups (CM1, CM2, and CM3). The detailed formulations are provided in Supplementary Table S1. The control group WM4 was formulated with wheat straw as the primary carbon source, following a commercial standard cultivation formulation [19]. In Phase I, the compost was turned 3–4 times, with peak temperatures maintained at 70–80 °C. In Phase II, the compost underwent pasteurization at 56–60 °C to eliminate contaminant microorganisms and pests, followed by temperature control at 45–48 °C for ammonia removal. After completion of Phase II, the compost was cooled to 25 °C for inoculation. *Agaricus bisporus* grain spawn strain A15, obtained from Sylvan Company, was used for inoculation. Post-inoculation, the substrate temperature was maintained at 25 °C to promote mycelial growth.

Upon complete mycelial colonization, a casing soil layer was applied uniformly at a uniform thickness of 3–5 cm across the surface. A small portion of fully colonized compost was incorporated into the casing layer (CACing). When mycelia had completely established throughout the casing soil surface, the temperature was reduced to 19–21 °C to initiate fruiting body management. Mushroom fruiting bodies measuring 3–5 cm were randomly sampled from the first flush. Fresh samples were immediately used for electronic tongue analysis, while portions were preserved at −80 °C for 16S amplicon sequencing and untargeted metabolomics experiments. Additional samples were dried to constant weight at 60 °C in an oven for compositional analysis. The yield was recorded as the total weight of fresh mushrooms harvested per square meter (kg/m^2^) across all flushes. Biological efficiency (BE) was calculated as the ratio of fresh mushroom yield to the dry weight of substrate, expressed as a percentage.

### 2.3. Determination of Chemical Components

#### 2.3.1. Proximate Composition Analysis

Crude protein levels were measured using the Kjeldahl method following AOAC (Association of Official Analytical Chemists) Official Method (1990) [20]. The Soxhlet extraction method was employed to determine the crude fat content. Ash content was evaluated using the dry ashing method. The crude polysaccharide content was determined using the water extraction-alcohol precipitation method and the phenol-sulfuric acid method. The crude fiber concentration was assessed following James’s protocol [21].

#### 2.3.2. Mineral Element Analysis

Dried samples (0.1 g) underwent acid digestion using 5 mL concentrated nitric acid and 2 mL hydrogen peroxide in digestion vessels. The solution was digested to clarity. Following cooling to room temperature, samples were diluted to a final volume of 25 mL with distilled water and subjected to filtration. Inductively Coupled Plasma Mass Spectrometry (ICP-MS) was utilized to quantify the mineral elements. Selenium concentration was measured separately by atomic fluorescence spectrometry to enhance analytical precision.

#### 2.3.3. Free Amino Acid Analysis

Dried sample powder (0.1 g) was combined with 0.02 mol/L HCl solution (5 mL) and homogenized to form a slurry. The mixture underwent sonication for 60 min, followed by centrifugal separation to obtain the supernatant, which was subsequently passed through a membrane filter. Subsequently, 200 µL of the supernatant was derivatized with 20 µL of n-norleucine (internal standard), 100 µL of triethylamine acetonitrile solution, and 100 µL of phenyl isothiocyanate (PITC) in acetonitrile solution in a 2 mL microcentrifuge tube. The reaction proceeded at room temperature (25 °C) for 60 min. Subsequently, 1 mL of n-hexane was introduced to the reaction mixture, which was then vigorously agitated and allowed to settle for 10 min to achieve phase separation. The aqueous phase was collected and subjected to a 5-fold dilution using distilled water. Before chromatographic analysis, the diluted samples were filtered using 0.22 µm syringe filters. High-performance liquid chromatography was conducted using an Amethyst C18-H column (250 mm × 4.6 mm, 5 µm particle size). All experimental procedures were carried out in triplicate to ensure reproducibility.

#### 2.3.4. 5′-Nucleotides Analysis

5′-Nucleotides were analyzed by HPLC using an Agilent 1200 system equipped with a DAD detector and a C18 column (4.6 mm × 250 mm, 5 µm). Samples were extracted with 10% perchloric acid under ultrasonic treatment for 30 min, neutralized to pH 6.5 with 5% KOH, filtered through a 0.22 µm membrane, and analyzed using phosphate buffer-methanol (1000:40, *v*/*v*) as mobile phase at 1.0 mL/min flow rate, 25 °C column temperature, and 254 nm detection wavelength with 10 µL injection volume. Nucleotide content was calculated using Equation (1):

(1) *W* = (*C* − *C*_0_) × *V* × *N*/*m*

where *W* represents the content (mg/kg); *C* and *C*_0_ are concentrations in the sample and blank (mg/L); *V* is the volume (mL); *N* refers to the dilution factor; and *m* is the sample mass (g).

### 2.4. Electronic Tongue Measurement

Taste characteristics were assessed employing the SA 402B taste sensing apparatus (Intelligent Sensor Technology, Inc., Kanagawa, Japan). Each sample (0.5 g) was thoroughly ground and dissolved in 100 mL of distilled water to extract taste compounds. The resulting mixture underwent centrifugation (8000× *g*, 15 min), after which the supernatant fraction was retrieved for measurement [22].

### 2.5. TAV and EUC Values Analysis

The taste activity value (*TAV*) was determined through Equation (2):

(2) *TAV* = *C*/*T*

where *C* denotes absolute concentration (mg/100 g), and *T* represents the established taste threshold values. *TAV* ≥ 1 indicated a significant taste contribution.

The equivalent umami concentration (*EUC*) was calculated using Equation (3):

(3) *EUC* = Σ*a*_i_*b*_i_ + 1218 (Σ*a*_i_*b*_i_) (Σ*a*_j_*b*_j_)

in which *a_i_* and *a_j_* denote the content levels of umami-contributing amino acids (aspartic acid and glutamic acid) and 5′-nucleotides (AMP, IMP, and GMP), respectively. The parameters *b*_i_ and *b*_j_ represent the corresponding relative intensity coefficients, with values of 0.077 for Asp, 1.000 for Glu, 0.18 for AMP, 1.00 for IMP, and 2.30 for GMP. The constant 1218 serves as the synergistic enhancement coefficient. Results were expressed as the amount of sodium glutamate (MSG) per 100 g sample [23].

### 2.6. Untargeted Metabolomics Analysis

Untargeted metabolomics analysis was conducted using a Vanquish UHPLC system (Thermo Fisher, Bremen, Germany) coupled with an Orbitrap Q Exactive^TM^ HF-X mass spectrometer (Thermo Fisher, Bremen, Germany). For polar compounds, samples were separated on a Hypersil Gold column (100 mm × 2.1 mm, 1.9 µm) with a 12 min linear gradient (0.2 mL/min flow rate). Mass spectrometry was performed in both positive and negative ionization modes, using optimized parameters including a 3.5 kV spray voltage, a 320 °C capillary temperature, and a 350 °C auxiliary gas heater temperature. Raw data were imported into XCMS software for peak alignment, detection, and quantification. Metabolite identification was achieved by comparing accurate masses (10 ppm tolerance) and MS/MS fragmentation patterns against secondary spectral databases. Each peak intensity was normalized relative to the total spectral intensity and subjected to standardization prior to statistical evaluation. Unsupervised principal component analysis (PCA) and supervised orthogonal partial least squares discriminant analysis (OPLS-DA) were performed using the ropls package in R (v4.3.1) to visualize sample clustering and discriminate between experimental groups. Model quality was evaluated using the R^2^X, R^2^Y, and Q^2^ parameters obtained through 7-fold cross-validation, and model robustness against overfitting was assessed using a 200-permutation test. Differential metabolites were identified using two-tailed Student’s *t*-tests combined with Benjamini–Hochberg (BH) false discovery rate (FDR) correction, applying thresholds of |fold change (FC)| > 2 and FDR-adjusted *p* < 0.05; the variable importance in projection (VIP) score (VIP > 1) derived from the OPLS-DA model was used as an auxiliary screening criterion. For pathway analysis, metabolites were annotated based on the KEGG database [24], and pathway enrichment analysis was performed using the MetaboAnalystR package (v2.0.1) with the generic KEGG reference pathway library; the background metabolite set corresponded to the complete set of KEGG compounds included in this default reference library. Over-representation analysis (ORA) was conducted using the hypergeometric test, with both raw *p*-values and BH-FDR-adjusted *p*-values reported, and pathway impact values were calculated based on topological analysis of the corresponding compound reaction network.

### 2.7. High-Throughput Sequencing

The cetyltrimethylammonium bromide (CTAB) procedure was employed for extracting genomic DNA. The primer pair 341F/806R was employed to amplify the bacterial 16S rRNA gene (V3-V4 region) [25]. Following verification by agarose gel electrophoresis, PCR products were sequenced on the Illumina sequencing platform. Raw sequence data underwent quality control, denoising, merging, and chimera removal using QIIME2 (2022.2), with amplicon sequence variants (ASVs) generated using the DADA2 plugin. Alpha diversity metrics were determined using QIIME2, whereas beta diversity analyses were conducted in R software (version 4.4.0).

### 2.8. Statistical Analysis

Conventional nutritional and sensory (electronic tongue) indices were determined with three biological replicates (*n* = 3), untargeted metabolomics analysis was performed with six biological replicates (*n* = 6), and 16S rRNA sequencing was performed with three biological replicates (*n* = 3). Data are expressed as mean ± standard deviation (SD). Statistical analyses were performed using SPSS Statistics 29.0, Microsoft Excel 2021, and R 4.3.1. Group comparisons were performed using one-way analysis of variance (ANOVA) followed by Tukey’s honestly significant difference (HSD) post hoc test (α = 0.05). When the assumption of homogeneity of variance was violated, Welch’s ANOVA followed by the Games-Howell post hoc test was applied instead; when data did not satisfy the assumption of normality, the Kruskal–Wallis H test was used, followed by Dunn’s post hoc test with BH-FDR correction for pairwise comparisons. *p*-values obtained from metabolomics and other multivariate analyses were corrected for multiple testing using the Benjamini–Hochberg (BH) false discovery rate (FDR) method. Pairwise associations among continuous phenotypic indices were evaluated using Pearson correlation analysis. Mantel tests were performed using the linkET package in R to assess associations between sensory attributes and taste-active compounds; Euclidean distance and Bray–Curtis distance matrices were constructed for the two data types, respectively, and statistical significance was determined based on Spearman’s correlation coefficients with 999 permutations. Partial least squares discriminant analysis (PLS-DA) was performed using the ropls package in R to identify taste markers, with all variables subjected to unit-variance scaling (auto-scaling) to eliminate differences in measurement scale. Model fit was evaluated using the R^2^X, R^2^Y, and Q^2^ parameters obtained through 7-fold cross-validation, and model stability was verified using a 200-permutation test to exclude the risk of overfitting. Taste markers were defined as variables satisfying both a VIP > 1 threshold in the PLS-DA model and a BH-FDR-corrected *p* < 0.05 in one-way ANOVA. For the PCA-based comprehensive evaluation, all raw indices were standardized using Z-score transformation, and principal components with eigenvalues greater than 1 were retained. Linear combination coefficients were derived from the principal component loading matrix, and the weighting coefficient of each retained principal component was calculated as the ratio of its variance contribution rate to the cumulative variance contribution rate of all retained components. These weights were used to compute a composite score for each sample.

## 3. Results and Discussion

### 3.1. Yield and Nutrient Composition

#### 3.1.1. Yield and Proximate Composition of *Agaricus bisporus* Cultivated on Different Substrates

The yield and proximate composition of mushrooms cultivated on various substrates were analyzed (Table 1). The yield of mushrooms cultivated on corn stover-based substrates ranged from 20.56 to 25.63 kg/m^2^, with biological efficiency values between 80.32% and 88.58%. The wheat straw-based treatment (WM4) achieved a yield of 24.23 kg/m^2^, which did not differ significantly from those of the three corn stover treatments (CM1, CM2, CM3). Its biological efficiency (91.35%) was significantly higher than that of CM3 (*p* < 0.05), but not significantly different from CM1 or CM2. While mushrooms grown on corn stover substrates generally showed high crude protein levels (30.21–33.81 g/100 g), CM2 and CM3 were significantly higher than those grown on the wheat straw substrate (WM4, 28.24 g/100 g; *p* < 0.05). Mushroom protein content is significantly influenced by the biological and chemical characteristics of the substrate [26,27]. Higher ash content was observed in CM2 (10.15 g/100 g) compared to 9.32 g/100 g in WM4. The crude polysaccharide content was higher in WM4 than in CM3. The crude fat and crude fiber contents showed no significant differences among treatments.

#### 3.1.2. Mineral Elements in *Agaricus bisporus* Cultivated on Different Substrates

Different substrate formulations significantly influenced the mineral element contents in *A. bisporus*. Compared to the traditional wheat straw and chicken manure cultivation substrate (WM4), corn stover substrates markedly altered the mineral element composition of *A. bisporus* (Table 2). CM1 exhibited high K (49.79 g/kg) and P (15.46 g/kg) contents, though they were not significantly different from WM4 and CM2, respectively (*p* > 0.05). Conversely, the CM2 treatment demonstrated optimal performance in the accumulation of Ca (0.38 ± 0.01 g/kg) and Mg (1.32 ± 0.03 g/kg). Sodium content was highest in WM4.

The effects of different substrate formulations on microelement contents in *A. bisporus* are presented in Table 2. All corn stover and chicken manure treatments resulted in significantly higher concentrations of Cu, Fe, and Zn in the mushrooms compared to the wheat straw treatment (*p* < 0.05). However, Se showed only partial differences among treatments, and Cr concentrations did not differ significantly (*p* > 0.05).

The variations in mineral profiles observed between corn stover- and wheat straw-based substrates can be attributed to substrate-dependent nutrient availability. As noted by several investigators, the nutritional composition of mushroom fruiting bodies is predominantly governed by substrate characteristics, which not only determine elemental content but also influence the accumulation of bioactive compounds and other nutrients, thereby enhancing their nutritional value [28,29,30]. Furthermore, the selection of agricultural residues and agro-industrial by-products as substrate components has been shown to substantially alter the mineral composition of cultivated mushrooms [31].

### 3.2. The Taste Compounds in Agaricus bisporus Cultivated on Different Substrates

#### 3.2.1. 5′-Nucleotides in *Agaricus bisporus* Cultivated on Different Substrates

As shown in Supplemental Table S2, only three nucleotides (5′-AMP, 5′-UMP, and 5′-GMP) were detected across all treatment groups, while 5’-CMP and 5’-IMP were not detected. Among all flavor nucleotides, 5’-AMP exhibited the highest content with significant differences between groups (*p* < 0.05). The WM4 treatment (wheat straw and chicken manure) has the highest 5’-AMP content at 586.79 ± 6.99 mg/kg, which is significantly higher than that of all other treatments. In contrast, 5’-UMP and 5’-GMP contents followed an opposing trend, both peaking in CM3 (419.42 ± 45.85 and 114.05 ± 1.69 mg/kg, respectively) and lowest in WM4 (79.42 ± 4.08 and 47.44 ± 0.64 mg/kg, respectively). Therefore, different cultivation substrates exert distinct regulatory effects on the accumulation of various flavor nucleotides in *A. bisporus*. Wheat straw substrates are more conducive to the accumulation of 5’-AMP, while corn stover substrates showed the highest levels for 5’-UMP and 5’-GMP accumulation. Among these nucleotides, 5’-GMP is particularly noteworthy, as it serves as an effective flavor enhancer that contributes to umami taste generation.

#### 3.2.2. Free Amino Acid and EUC in *Agaricus bisporus* Cultivated on Different Substrates

Table 3 presents an analysis of free amino acid composition in *A. bisporus* cultivated on different substrates. Among the 16 amino acids detected, Ala, Glu, Leu, Val, and Pro exhibited the highest concentrations across all treatments, while Tyr, Met, and His showed relatively low levels. Compared to the wheat straw treatment (WM4), all three corn stover treatments (CM1, CM2, and CM3) exhibited significantly higher contents of most free amino acids, except Arg, Thr, Tyr, and Met.

To evaluate potential taste characteristics, amino acids were categorized into four groups: umami amino acids (UAC), sweet amino acids (SAC), bitter amino acids (BAC), and tasteless amino acids (TAC) (Table 3) [32]. All corn stover treatments exhibited significantly higher UAC content than the wheat straw treatment (*p* < 0.05), with the CM2 treatment achieving the highest UAC content. Research by Beluhan and Ranogajec demonstrated that the synergistic effect of aspartic acid and glutamic acid generates MSG-like or savory flavors. MSG-like components also influence the EUC levels in mushrooms; mushrooms with high concentrations of MSG-like compounds tend to exhibit elevated EUC values [33]. In one comparative study involving 17 edible mushroom species, *A. bisporus* demonstrated the highest MSG-like component content (42.4 ± 6.90 mg/g) [5]. All corn stover treatments showed significantly higher SAC contents than the wheat straw treatment. Regarding BAC, CM1 exhibited the highest content (9.72 ± 0.02 mg/g), followed by CM3 (9.53 ± 0.04 mg/g), CM2 (7.48 ± 0.04 mg/g), and the wheat straw treatment (6.69 ± 0.02 mg/g). The TAC content in corn stover treatments was also significantly higher than in the wheat straw treatment, with CM3 showing the highest TAC content (1.23 ± 0.02 mg/g).

Overall, all corn stover treatments exhibited significantly higher total amino acid (TAA) contents than the wheat straw treatment, with CM3 achieving the highest content (36.15 ± 0.14 mg/g), followed by CM1 (34.61 ± 0.05 mg/g) and CM2 (31.42 ± 0.18 mg/g). These results indicate that corn stover substrates, particularly the CM3 treatment, can significantly enhance the accumulation of flavor-related amino acids in fruiting bodies compared to wheat straw substrates.

To comprehensively evaluate the overall umami characteristics of the samples, we employed EUC values for analysis. The EUC analysis revealed that the umami intensity of the four treatments ranked in descending order as follows: CM3 > CM2 > CM1 > WM4. Significant differences were detected between all treatments (*p* < 0.05). The EUC value of the CM3 treatment was 28.93 g MSG/100 g, while that of the WM4 treatment was 13.41 g MSG/100 g. Both values fell within Level 2 (10–100 g MSG/100 g) of Mau’s classification system, indicating moderate umami intensity [34]. These values were within the range of umami intensities reported for edible mushrooms in the literature [5,9,32]. Notably, the corn stover-based substrates generally resulted in higher EUC values than the wheat straw substrate, indicating an overall enhancement of umami intensity, which aligns with the enhancement trend observed in studies on *Lentinula edodes* cultivation, where rice straw was used as a substrate substitute, with an 8.1-fold increase documented (EUC increased from 10.42 to 84.49 g MSG/100 g) [35].

#### 3.2.3. Variation in TAV

TAVs were relatively high in the corn stover treatments (57.96–64.07), but lower in the wheat straw treatment WM4 (44.91). The primary free amino acids contributing to taste were Glu (16.27–25.60, umami), Ala (12.02–16.82, sweet), Val (3.90–5.68, bitter), and Arg (1.52–3.04, bitter). These compounds contributed substantially to taste in corn stover treatments but showed reduced activity in the wheat straw treatment. Glu peaked at CM2 (25.60), Ala and Val at CM1 (16.82 and 5.68), while Arg and His reached their highest levels at CM3 (3.04 and 2.30). Pro was active only in CM3 (TAV = 1.08). Among the 5′-nucleotides, 5’-AMP exerted the strongest effect, active solely in WM4 (TAV = 1.17). In contrast, Asp, Ser, Gly, Thr, Tyr, Met, 5’-AMP, and 5’-GMP in corn stover treatments contributed minimally to the taste of *A. bisporus* (Supplemental Table S3).

### 3.3. Taste Traits by Electronic Tongue

**Figure 1:**
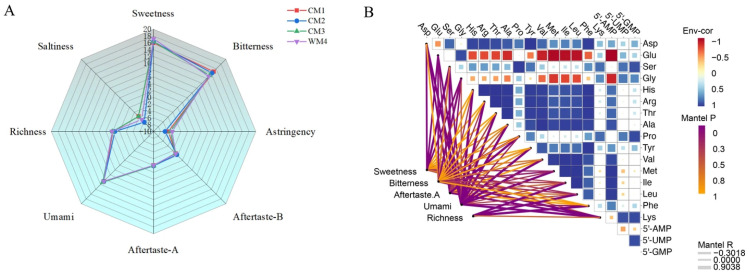
Relationship between non-volatile components and taste characteristics. (**A**) Radar charts displaying electronic tongue sensor response data. (**B**) The results of the correlation analysis.

The taste characteristics of *A. bisporus* fruiting bodies cultivated on different substrates were determined using an electronic tongue system, and the results are presented in Figure 1A. The electronic tongue system measured eight key taste parameters, including sweetness, bitterness, astringency, bitter aftertaste, astringent aftertaste, umami, richness, and saltiness. In terms of umami, the CM3 treatment showed the highest value (11.08), slightly higher than that of the wheat straw treatment (10.69), suggesting an enhancement in umami perception consistent with the increased EUC values. Mushrooms cultivated on corn stover substrates also showed higher bitterness responses. This moderate enhancement in bitterness aligns with the elevated BAC contents quantified in the corn stover treatments (Table 3). It should be noted that mushroom flavor also depends on volatile compounds—most notably C8 compounds such as 1-octen-3-ol—which were not assessed in the present study [36]. As comprehensive volatile profiling (e.g., GC-MS or electronic nose analysis) and human sensory panels lie beyond the current scope, and given that culinary preparation can further alter perceived aroma, the present findings should be regarded as characterizing taste rather than the complete flavor profile of *A. bisporus*. Future studies adopting an integrated “Sensomics” framework—combining instrumental volatilomics, non-volatile taste analysis, and human sensory evaluation—would offer a more comprehensive understanding of how cultivation substrate shapes overall flavor perception [37].

### 3.4. Correlation Analysis of Taste Components and Taste Sensation

This study evaluated the relationships between electronic tongue taste sensor responses (sweetness, bitterness, aftertaste-A, umami, and richness) and major non-volatile taste-active substances in *A. bisporus* fruiting bodies (Figure 1B). The bitter amino acids (Arg, His, Ile, Leu, Met, Phe, and Val) were positively correlated with one another and with the sweet amino acids Ala and Thr, and with the tasteless amino acid Tyr, while showing negative correlations with Gly. Among the umami amino acids, Asp was positively correlated with the bitter amino acids, whereas Glu was negatively correlated with them. Among the 5′-nucleotides, 5′-AMP was positively correlated with the bitter amino acids but negatively correlated with Glu, while 5′-UMP and 5’-GMP were positively correlated with each other and with most of the umami, sweet, and tasteless amino acids. This correlation analysis of electronic tongue response values and taste substances revealed complex interactions between taste perception and taste compounds. Richness showed strong positive correlations with most amino acids, particularly with Arg, His, Thr, Ala, and Val, but showed no significant correlations with 5’-UMP and 5’-GMP (r values of −0.048 and 0.102, respectively, *p* > 0.05). Furthermore, umami showed potential associations with free amino acids and 5’-nucleotides, showing the strongest correlation with 5’-GMP, followed by Pro, Ser, and Lys. Bitterness exhibited an extremely strong positive correlation with 5’-GMP and also showed significant positive correlations with Pro, Ser, Lys, and 5’-UMP. Aftertaste intensity was primarily correlated with 5’-UMP, and also displayed significant positive correlations with Gly, 5’-GMP, and Lys (r values ranging from 0.559 to 0.698).

### 3.5. Differential Analyses of PLS-DA on Nutrition and Taste

**Figure 2:**
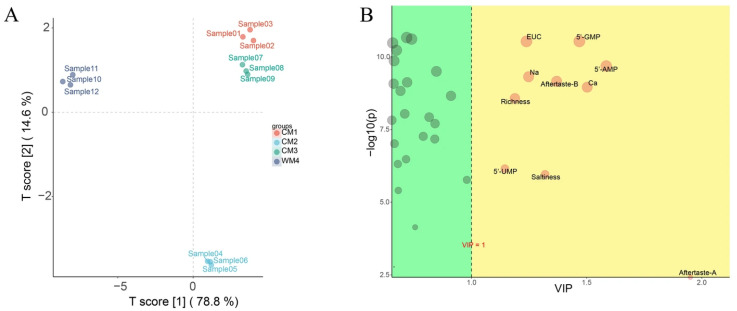
PLS-DA for non-volatile components, nutritional compounds, and electronic tongue taste parameters. PLS-DA score plot (**A**). The VIP values from PLS-DA (**B**).

The partial least squares discriminant analysis (PLS-DA) score plot (Figure 2A) illustrated the distribution of samples from each treatment in two-dimensional space, with component 1 (explaining 78.8% of variance) and component 2 (explaining 14.6% of variance) on the horizontal and vertical axes, respectively, cumulatively accounting for 93.4% of the total variance. Samples from CM1, CM2, CM3, and WM4 treatments demonstrated distinct separation patterns, indicating clear separation among treatments in nutritional composition and taste characteristics across different cultivation substrates. The PLS-DA model identified 10 key markers (Variable Importance in Projection (VIP) > 1, *p* < 0.05) that significantly differentiate the nutritional and flavor profiles of *A. bisporus* across different substrates (Figure 2B), ranked by descending VIP values: aftertaste-A (1.950), 5′-AMP (1.585), Ca (1.502), 5′-GMP (1.468), aftertaste-B (1.369), saltiness (1.319), Na (1.248), EUC (1.238), richness (1.188), and 5′-UMP (1.145).

### 3.6. Comprehensive Evaluation

The comprehensive assessment of each treatment was performed using formula (Equations (4)–(7)), where the variables *X*_1_ to *X*_7_ represent the standardized values of crude protein, crude polysaccharides, macro-minerals, micro-minerals, total amino acids (TAA), total 5′-nucleotides, and umami, respectively:

(4) *Y*_1_ = 0.437*X*_1_ − 0.427*X*_2_ − 0.278*X*_3_ + 0.347*X*_4_ + 0.430*X*_5_ + 0.279*X*_6_ + 0.407*X*_7_

(5) *Y*_2_ = 0.293*X*_1_ + 0.231*X*_2_ − 0.014*X*_3_ + 0.556*X*_4_ + 0.191*X*_5_ − 0.644*X*_6_ − 0.316*X*_7_

(6) *Y*_3_ = − 0.286*X*_1_ − 0.318*X*_2_ + 0.710*X*_3_ + 0.234*X*_4_ + 0.382*X*_5_ + 0.191*X*_6_ − 0.276*X*_7_

(7) *Y* = 0.584 × *Y*_1_ + 0.219 × *Y*_2_ + 0.197 × *Y*_3_

Principal component analysis results demonstrated that CM3 and CM2 samples achieved the highest comprehensive evaluation scores of 1.4090 and 0.2237, respectively. In contrast, CM1 and WM4 samples exhibited the lowest comprehensive evaluation scores of 0.1577 and −1.7903, respectively (Supplemental Table S4). The overall assessment revealed that corn stover substrates consistently demonstrated superior nutritional characteristics compared to the wheat straw treatment. These findings provide scientific evidence for mushroom production and processing, indicating that optimization of cultivation conditions can significantly enhance the nutritional value and quality attributes of mushroom products.

### 3.7. Non-Targeted Metabolomics Analysis

#### 3.7.1. Composition of Major Metabolites

**Figure 3:**
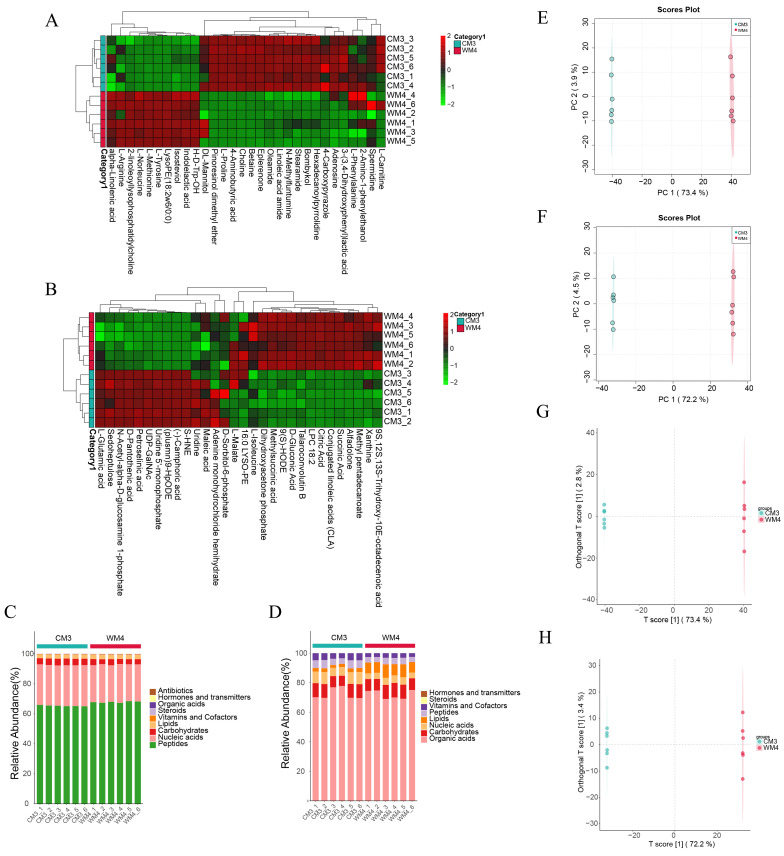
Changes in metabolites of *Agaricus bisporus* cultivated on corn stover and wheat straw substrates. Heatmaps display the top 30 metabolites in ESI+ mode (**A**) and ESI– mode (**B**). Relative abundance of metabolite classifications in ESI+ mode (**C**) and ESI– mode (**D**). Principal component analysis (PCA) score plots in ESI+ mode (**E**) and ESI– mode (**F**). OPLS-DA score plots in ESI+ mode (**G**) and ESI– mode (**H**). CM3: mushrooms cultivated on corn stover substrate; WM4: mushrooms cultivated on wheat straw substrate.

For untargeted metabolomics analysis, CM3 and WM4 were selected as representative samples. A total of 3997 metabolic features were detected. Figure 3A,B illustrate the abundance of the top 30 compounds detected under positive ion mode (ESI+) and negative ion mode (ESI−), respectively. Under ESI+ mode, metabolites in *A. bisporus* were categorized into nine classes. Compared to *A. bisporus* cultivated on wheat straw substrates, mushrooms grown on corn stover substrates exhibited increased levels of nucleic acids, carbohydrates, and vitamins and cofactors (Figure 3C). Under ESI− mode, *A. bisporus* grown on corn stover demonstrated elevated levels of organic acids, nucleic acids, peptides, vitamins and cofactors, steroid compounds, as well as hormones and transmitters, while exhibiting reduced concentrations of carbohydrates and lipid compounds (Figure 3D). The distinct global metabolic profiles of *A. bisporus* cultivated on different substrates are likely driven by the inherent variations in raw material composition, which determine the quality and quantity of bioavailable nutrients available to the mushrooms following microbial degradation during the composting and cultivation processes. Consistently, the observed metabolic patterns correspond to measured differences in crude protein, free amino acids, and other nutritional and taste components, highlighting the influence of substrate type on metabolic networks and on the nutritional and taste composition of the fruiting bodies. These substrate-driven metabolic responses are consistent with previous findings in other mushroom species. In *Pleurotus ostreatus* cultivated on basal substrates supplemented with *Astragalus membranaceus* var. *mongolicus* (AMM) waste, amino acids and peptides, steroids, phenolics, fatty acids, and benzoic acids represented the most bioactive metabolite classes [38].

#### 3.7.2. Multivariate and Univariate Analysis

Unsupervised principal component analysis (PCA) revealed clear separation between *A. bisporus* cultivated on corn stover versus wheat straw. In ESI+ mode, PC1 and PC2 explained 73.4% and 3.9% of the variance, respectively; in ESI− mode, they explained 72.2% and 4.5%, respectively (Figure 3E,F). Orthogonal partial least-squares discriminant analysis (OPLS-DA) further confirmed distinct metabolic profiles (Figure 3G,H). Characteristic metabolites were identified based on univariate analysis. Using selection criteria of absolute fold change > 2 and adjusted *p* < 0.05, a total of 593 differential metabolites were identified in the experimental group (CM3) compared to the control group (WM4) in ESI+ mode (278 upregulated, 315 downregulated). Meanwhile, only 375 metabolites were identified in ESI− mode (178 upregulated, 197 downregulated) (Figure S1).

In ESI+ mode (Figure S1A), when comparing *A. bisporus* cultivated on corn stover with that cultivated on wheat straw, the major upregulated metabolites primarily included amino acids, peptides, and their analogs (L-proline, Lys Leu Ser), as well as lipids and lipid-like molecules (9(S)-HOT). These amino acid-related metabolites are involved in protein synthesis and nitrogen metabolism. Previous metabolomics studies have shown that different cultivation substrates significantly altered the expression profiles of amino acids and dipeptides in *Pleurotus ostreatus*, suggesting that substrate selection may represent a strategy to modulate potentially bioactive components in mushrooms [39]. Additionally, lipid-related metabolites are associated with unsaturated fatty acid metabolism. On the other hand, downregulated metabolites in CM3 included amino acids and derivatives (L-Tyrosine, L-phenylalanine-L-proline, and L-isoleucine-L-proline) as well as lipids or related compounds. In ESI− mode (Figure S1B), differentially expressed metabolites primarily included lipids and lipid-like molecules, and organic acids and derivatives (gamma-L-Glutamyl-L-methionine sulfoxide).

#### 3.7.3. Metabolic Pathway Enrichment Analysis

**Figure 4:**
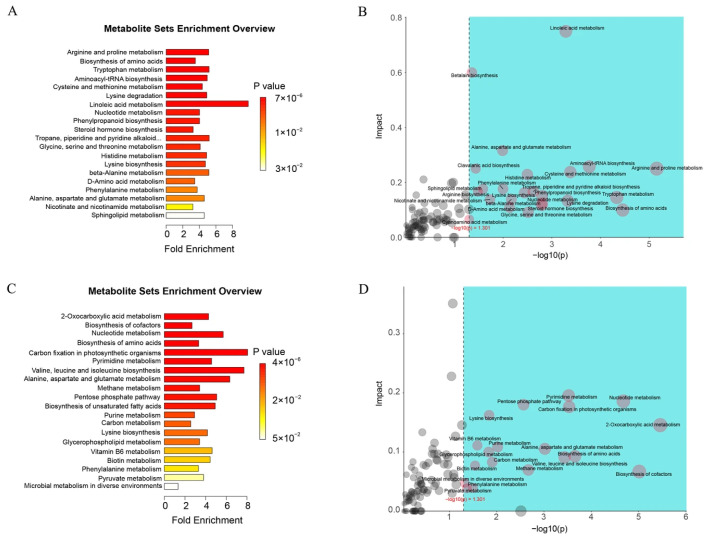
Prediction of metabolic pathways through identification of significantly differential metabolites in *Agaricus bisporus* cultivated on corn stover and wheat straw. Over-representation analysis (ORA) enrichment analysis in ESI+ mode (**A**) and ESI– mode (**C**). Color intensity indicates the magnitude of the *p*-value, with darker colors corresponding to smaller *p*-values. Integration of ORA enrichment analysis and topological analysis in ESI+ mode (**B**) and ESI– mode (**D**). The horizontal axis represents the *p*-value from ORA, with metabolic pathways in the blue region being significant (*p* < 0.05) in the ORA enrichment analysis. The vertical axis indicates the impact of these metabolic pathways in the topological analysis.

The metabolic pathways associated with differential metabolites from *A. bisporus* cultivated on corn stover and wheat straw substrates were systematically analyzed (Figure 4). The differentially accumulated metabolites between CM3 and WM4 were predominantly enriched in interconnected pathways governing amino acid and nitrogen metabolism, including arginine and proline metabolism, cysteine and methionine metabolism, tryptophan metabolism, and biosynthesis of amino acids. These enrichments directly correspond to the observed differences in crude protein content and free amino acid profiles between corn stover- and wheat straw-cultivated *A. bisporus* (Table 1 and Table 3), providing a mechanistic basis for substrate-dependent variations in nitrogenous compound accumulation.

Several pathways emerging from the ORA results—notably “carbon fixation in photosynthetic organisms,” “clavulanic acid biosynthesis,” and “tropane, piperidine and pyridine alkaloid biosynthesis”—are not biosynthetically plausible in *A. bisporus*: this fungus is a non-photosynthetic basidiomycete that possesses neither β-lactam biosynthetic machinery nor plant-type alkaloid biosynthetic pathways. Tracing the metabolites driving the enrichment of these pathways confirms that they derive from the mapping of shared primary intermediates to generic KEGG reference pathway categories, rather than from genuine pathway activity. Specifically, the enrichment of “carbon fixation in photosynthetic organisms” was driven entirely by central carbon metabolism intermediates (e.g., D-erythrose 4-phosphate, glycerone phosphate, and D-fructose 1,6-bisphosphate), which are also constituents of glycolysis and the pentose phosphate pathway; “clavulanic acid biosynthesis” was driven by L-arginine (not statistically significant); and “tropane, piperidine and pyridine alkaloid biosynthesis” was driven by shared amino-acid precursors such as L-lysine and L-phenylalanine—all of which are among the differential metabolites identified in this study. Because KEGG reference maps are constructed across taxa, these ubiquitous intermediates are simultaneously assigned to multiple lineage-specific pathways, causing over-representation analysis to flag pathways that are not actually present in the target organism [40]. We therefore regard these enrichment results as annotation-mapping artifacts and do not assign them any biological interpretive weight. By contrast, significant enrichment was also evident in nucleotide metabolism, as well as in central carbon metabolism pathways, notably 2-oxocarboxylic acid metabolism. These pathways provide critical carbon skeletons and energetic intermediates essential for nucleotide biosynthesis, consistent with the observed differences in 5′-nucleotide levels (Supplementary Table S2) and the umami perception of mushrooms cultivated on the two substrates.

The observed metabolic alterations can be attributed to the distinct lignocellulosic compositions and C/N ratios of corn stover and wheat straw [18,41]. The structural and chemical differences in the cellulose–hemicellulose–lignin complexes of these two agricultural residues result in differential degradability and carbon bioavailability for *A. bisporus* mycelium. Enhanced carbon availability likely promotes activation of carbohydrate metabolic pathways [42], subsequently providing more carbon skeletons and energy for amino acid biosynthesis and nucleotide synthesis [43]. This differential pathway activation underscores complex interactions between substrate-derived nutrients and the metabolic network of *A. bisporus*, ultimately influencing its nutritional quality and the accumulation of flavor-related metabolites.

### 3.8. Bacterial Diversity

**Figure 5:**
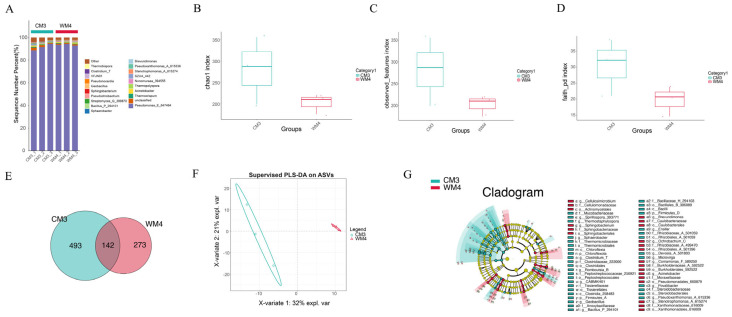
Changes in bacterial community composition within *Agaricus bisporus* fruiting bodies. (**A**) Changes in the relative abundance of bacterial communities at the genus level. (**B**–**D**) Bacterial alpha diversity. (**E**) Venn diagram. (**F**) PLS-DA plot of bacterial communities. (**G**) Biomarker analysis. CM3: mushrooms cultivated on corn stover substrate; WM4: mushrooms cultivated on wheat straw substrate.

The 16S rRNA amplicon sequencing analysis of mushroom fruiting bodies from CM3 and WM4 treatments revealed differences in bacterial community composition (Figure 5). At the genus level, *Pseudomonas* emerged as the predominant bacterial taxon in *A. bisporus* fruiting bodies cultivated on both corn stover (CM3) and wheat straw (WM4) substrates, accounting for over 90% of the relative abundance. This dominance of *Pseudomonas* in *A. bisporus* fruiting bodies cultivated on both substrates is consistent with previous findings [44,45]. The high abundance of *Pseudomonas* may be related to its chemotactic response to *A. bisporus* mycelial secretions and its rapid attachment capabilities [46,47]. Notably, although *Pseudomonas* dominated in both substrates, the corn stover treatment exhibited modestly higher overall alpha diversity, primarily through increased richness of rare, low-abundance taxa. This suggests that the corn stover substrate may provide limited additional ecological niche space for other microbial taxa, though the biological significance of this modest increase requires further functional validation. Venn diagram analysis revealed 493 unique ASVs in the CM3 treatment, 273 unique ASVs in the WM4 treatment, and 142 shared ASVs between the two treatments.

The microbial community structure of the CM3 and WM4 treatments differed significantly, as shown by PLS-DA analysis. The two treatments formed distinctly separated clusters in the ordination plot, indicating that different cultivation substrates exerted substantial influence on the microbial community composition of *A. bisporus* fruiting bodies. Linear discriminant analysis Effect Size (LEfSe) identified statistically significant biomarkers distinguishing the two treatments.

## 4. Conclusions

This study compared the yield, biological efficiency, nutritional composition, mineral profile, taste-active compounds, metabolomic characteristics, and bacterial communities of *Agaricus bisporus* cultivated on corn stover- and wheat straw-based substrates. The corn stover-based substrates supported the efficient cultivation of *A. bisporus*, with yields of 20.56–25.63 kg/m^2^, not significantly different from the wheat straw-based substrate (WM4, 24.23 kg/m^2^). Biological efficiency ranged from 80.32% to 88.58%; CM1 and CM2 were comparable to WM4 (91.35%), whereas CM3—the treatment with the most favorable nutritional and umami quality—showed significantly lower biological efficiency than WM4 (*p* < 0.05). In terms of nutrient composition, wheat straw-based substrate was found to be more favorable for crude polysaccharides and 5′-AMP accumulation, whereas corn stover-based substrates delivered higher crude protein, mineral elements (Cu, Fe, and Zn), and enhanced umami intensity as evidenced by elevated EUC and electronic tongue profiles. Metabolomics (CM3 vs. WM4) revealed significant enrichment in nucleotide metabolism and amino acid biosynthesis pathways, while bacterial communities remained dominated by *Pseudomonas*. This work highlights corn stover as a viable resource for agricultural residue utilization and fundamentally advances our understanding of substrate-dependent nutritional and flavor modulation in edible mushrooms. It should be noted that the quality–biological efficiency trade-off observed in CM3 is an important consideration when selecting corn stover formulations for practical production.

## Figures and Tables

**Table 1:** Yield, biological efficiency, and proximate composition of *Agaricus bisporus* cultivated on corn stover- and wheat straw-based substrates.

Treatment Group	Yield (kg/m^2^)	BE (%)	Crude Protein (g/100 g)	Ash (g/100 g)	Crude Fat (g/100 g)	Crude Fiber (g/100 g)	Crude Polysaccharides (g/100 g)
CM1	23.00 ± 2.25 ^ab^	86.12 ± 2.99 ^ab^	30.21 ± 1.09 ^bc^	9.62 ± 0.11 ^b^	4.97 ± 0.06 ^a^	6.37 ± 0.21 ^a^	1.91 ± 0.06 ^a^
CM2	25.63 ± 0.67 ^a^	88.58 ± 2.31 ^ab^	33.00 ± 0.22 ^ab^	10.15 ± 0.14 ^a^	4.78 ± 0.20 ^a^	6.60 ± 0.44 ^a^	1.97 ± 0.04 ^a^
CM3	20.56 ± 1.63 ^b^	80.32 ± 4.36 ^b^	33.81 ± 1.64 ^a^	9.48 ± 0.26 ^b^	4.83 ± 0.14 ^a^	6.47 ± 0.15 ^a^	1.73 ± 0.08 ^b^
WM4	24.23 ± 1.14 ^ab^	91.35 ± 2.84 ^a^	28.24 ± 1.39 ^c^	9.32 ± 0.08 ^b^	4.95 ± 0.15 ^a^	6.33 ± 0.25 ^a^	2.03 ± 0.07 ^a^

Note: Values are expressed as mean ± standard deviation (SD) of three independent biological replicates (*n* = 3). Different letters in the same column indicate significant differences at *p* < 0.05 (one-way ANOVA followed by Tukey’s honestly significant difference (HSD) post hoc test for multiple comparisons).

**Table 2:** Mineral element contents in *A. bisporus* cultivated on different substrates.

	CM1	CM2	CM3	WM4
K (g/kg)	49.79 ± 1.15 ^a^	43.53 ± 0.12 ^b^	45.18 ± 2.26 ^b^	46.27 ± 1.56 ^ab^
Ca (g/kg)	0.09 ± 0.00 ^c^	0.38 ± 0.01 ^a^	0.09 ± 0.01 ^c^	0.29 ± 0.01 ^b^
Na (g/kg)	0.42 ± 0.01 ^c^	1.44 ± 0.03 ^b^	0.38 ± 0.03 ^c^	2.12 ± 0.06 ^a^
Mg (g/kg)	1.15 ± 0.03 ^b^	1.32 ± 0.03 ^a^	1.11 ± 0.07 ^bc^	1.03 ± 0.04 ^c^
P (g/kg)	15.46 ± 0.38 ^a^	15.17 ± 0.05 ^ab^	14.02 ± 0.53 ^c^	14.34 ± 0.33 ^bc^
B (mg/kg)	8.90 ± 0.62 ^a^	9.81 ± 0.69 ^a^	9.82 ± 0.62 ^a^	9.23 ± 0.59 ^a^
Cu (mg/kg)	30.32 ± 0.63 ^b^	34.75 ± 0.30 ^a^	36.32 ± 1.19 ^a^	17.36 ± 1.05 ^c^
Fe (mg/kg)	63.29 ± 1.57 ^a^	61.58 ± 1.68 ^a^	52.50 ± 1.66 ^b^	32.64 ± 2.45 ^c^
Zn (mg/kg)	31.81 ± 0.36 ^b^	34.93 ± 0.93 ^a^	35.80 ± 0.57 ^a^	24.99 ± 0.99 ^c^
Se (mg/kg)	2.37 ± 0.09 ^ab^	2.96 ± 0.91 ^a^	2.31 ± 0.09 ^ab^	1.52 ± 0.07 ^b^
Cr (mg/kg)	1.83 ± 0.52 ^a^	1.65 ± 0.27 ^a^	1.70 ± 0.29 ^a^	1.33 ± 0.01 ^a^

Note: Values are expressed as mean ± standard deviation (SD) of three independent biological replicates (*n* = 3). Different letters in the same row indicate significant differences at *p* < 0.05 (one-way ANOVA followed by Tukey’s honestly significant difference (HSD) post hoc test for multiple comparisons).

**Table 3:** The content of free amino acids and EUC in *Agaricus bisporus* cultivated on different substrates.

	CM1	CM2	CM3	WM4
Asp (mg/g)	0.78 ± 0.00 ^b^	0.72 ± 0.01 ^c^	0.82 ± 0.01 ^a^	0.70 ± 0.01 ^d^
Glu (mg/g)	6.04 ± 0.01 ^c^	7.68 ± 0.04 ^a^	7.03 ± 0.03 ^b^	4.88 ± 0.05 ^d^
Ser (mg/g)	1.33 ± 0.01 ^b^	1.32 ± 0.01 ^b^	1.42 ± 0.01 ^a^	1.09 ± 0.00 ^c^
Gly (mg/g)	0.81 ± 0.01 ^b^	0.96 ± 0.01 ^a^	0.96 ± 0.01 ^a^	0.63 ± 0.00 ^c^
His (mg/g)	0.44 ± 0.01 ^b^	0.28 ± 0.00 ^c^	0.46 ± 0.00 ^a^	0.26 ± 0.01 ^d^
Arg (mg/g)	1.42 ± 0.01 ^b^	0.76 ± 0.01 ^d^	1.52 ± 0.01 ^a^	0.78 ± 0.01 ^c^
Thr (mg/g)	1.86 ± 0.01 ^b^	1.03 ± 0.01 ^d^	1.93 ± 0.01 ^a^	1.35 ± 0.01 ^c^
Ala (mg/g)	10.09 ± 0.02 ^a^	8.26 ± 0.05 ^c^	9.99 ± 0.05 ^b^	7.21 ± 0.02 ^d^
Pro (mg/g)	2.85 ± 0.03 ^b^	2.85 ± 0.04 ^b^	3.24 ± 0.04 ^a^	1.67 ± 0.02 ^c^
Tyr (mg/g)	0.10 ± 0.00 ^b^	0.06 ± 0.00 ^d^	0.12 ± 0.00 ^a^	0.08 ± 0.00 ^c^
Val (mg/g)	2.27 ± 0.02 ^a^	1.93 ± 0.01 ^c^	2.22 ± 0.01 ^b^	1.56 ± 0.01 ^d^
Met (mg/g)	0.17 ± 0.00 ^b^	0.06 ± 0.00 ^d^	0.13 ± 0.00 ^c^	0.18 ± 0.01 ^a^
Ile (mg/g)	1.86 ± 0.01 ^a^	1.53 ± 0.01 ^c^	1.76 ± 0.02 ^b^	1.33 ± 0.01 ^d^
Leu (mg/g)	2.52 ± 0.01 ^a^	1.97 ± 0.01 ^c^	2.37 ± 0.02 ^b^	1.73 ± 0.01 ^d^
Phe (mg/g)	1.04 ± 0.02 ^b^	0.95 ± 0.02 ^c^	1.07 ± 0.01 ^a^	0.85 ± 0.01 ^d^
Lys (mg/g)	1.03 ± 0.01 ^c^	1.06 ± 0.01 ^b^	1.11 ± 0.01 ^a^	0.66 ± 0.02 ^d^
UAC (mg/g)	6.82 ± 0.01 ^c^	8.40 ± 0.03 ^a^	7.85 ± 0.03 ^b^	5.58 ± 0.06 ^d^
SAC (mg/g)	16.94 ± 0.02 ^b^	14.42 ± 0.12 ^c^	17.54 ± 0.11 ^a^	11.95 ± 0.05 ^d^
BAC (mg/g)	9.72 ± 0.02 ^a^	7.48 ± 0.04 ^c^	9.53 ± 0.04 ^b^	6.69 ± 0.02 ^d^
TAC (mg/g)	1.13 ± 0.01 ^b^	1.12 ± 0.01 ^b^	1.23 ± 0.02 ^a^	0.74 ± 0.03 ^c^
TAA (mg/g)	34.61 ± 0.05 ^b^	31.42 ± 0.18 ^c^	36.15 ± 0.14 ^a^	24.96 ± 0.07 ^d^
EUC (g MSG/100 g)	16.07 ± 0.10 ^c^	19.89 ± 0.21 ^b^	28.93 ± 0.44 ^a^	13.41 ± 0.03 ^d^

Note: Values are expressed as mean ± standard deviation (SD) of three independent biological replicates (*n* = 3). Different letters in the same row indicate significant differences at *p* < 0.05 (one-way ANOVA followed by Tukey’s honestly significant difference (HSD) post hoc test for multiple comparisons).

## Data Availability

The original contributions presented in this study are included in the article/Supplementary Material. Further inquiries can be directed to the corresponding authors.

## Supplementary Materials

The following supporting information can be downloaded at: https://www.mdpi.com/article/10.3390/foods15142570/s1. Table S1: The fomulation of different compost; Table S2: The 5′-nucleotides content in *Agaricus bisporus* cultivated on different substrates. Table S3: TAV of taste components in *Agaricus bisporus* cultivated on different substrates; Table S4: The scores of principal component factors and comprehensive evaluation score. Figure S1: Volcano plot analysis of metabolites with differential abundance between *Agaricus bisporus* cultivated on corn stover-based substrate (CM3) and wheat straw-based substrate (WM4). (A): ESI+ mode; (B): ESI− mode.
