# Supplementary material for: Nutritional, Mineral, Taste, and Metabolic Profiles of Agaricus bisporus Cultivated on Corn Stover Substrate: A High-Quality Alternative to Wheat Straw
Source: Foods. 2026 Jul 22;15(14):2570. doi: 10.3390/foods15142570 (PMC13407967; doi:10.3390/foods15142570)
Supplement: Supplementary file 1 [file foods-15-02570-s001.zip › foods-4441555-supplementary.pdf]

Supplementary **Table S1**

The fomulation of different compost.

	Corn stover	Chicken manure	Corn distillers' grains	Gypsum
CM1	54.35%	42.15%	2.00%	1.50%
CM2	59.79%	36.71%	2.00%	1.50%
CM3	65.16%	31.34%	2.00%	1.50%

Supplementary **Table S2**

The 5′-nucleotides content in *Agaricus bisporus* cultivated on different substrates.

	CM1	CM2	CM3	WM4
5'-CMP (mg/kg)	–	–	–	–
5'-AMP (mg/kg)	487.04 ± 2.96^b	205.36 ± 2.07^d	356.20 ± 3.92^c	586.79 ± 6.99^a
5'-UMP (mg/kg)	172.68 ± 52.41^c	290.76 ± 4.60^b	419.42 ± 45.85^a	79.42 ± 4.08^d
5'-GMP (mg/kg)	52.44 ± 0.47^c	72.10 ± 0.46^b	114.05 ± 1.69^a	47.44 ± 0.64^d
5'-IMP (mg/kg)	–	–	–	–

Note: Different letters in the same row indicate significant differences at $p < 0.05$.

Supplementary **Table S3**

TAV of taste components in *Agaricus bisporus* cultivated on different substrates.

Taste components	Threshold (mg/g in water)	TAV			
		CM1	CM2	CM3	WM4
Free amino acids					
Asp	1	0.78	0.72	0.82	0.70
Glu	0.3	20.13	25.60	23.43	16.27
Ser	1.5	0.89	0.88	0.95	0.73
Gly	1.3	0.62	0.74	0.74	0.48
His	0.2	2.20	1.40	2.30	1.30
Arg	0.5	2.84	1.52	3.04	1.56
Thr	2.6	0.72	0.40	0.74	0.52
Ala	0.6	16.82	13.77	16.65	12.02
Pro	3	0.95	0.95	1.08	0.56
Tyr	0.91	0.11	0.07	0.13	0.09
Val	0.4	5.68	4.83	5.55	3.90
Met	0.3	0.57	0.20	0.43	0.60
Ile	0.9	2.07	1.70	1.96	1.48
Leu	1.9	1.33	1.04	1.25	0.91
Phe	0.9	1.16	1.06	1.19	0.94
Lys	0.5	2.06	2.12	2.22	1.32
5′-Nucleotides					
5'-AMP	0.5	0.97	0.41	0.71	1.17
5'-GMP	0.13	0.40	0.55	0.88	0.36
Total	–	60.30	57.96	64.07	44.91

Supplementary **Table S4**

The scores of principal component factors and comprehensive evaluation score.

Treatment group	PC1 (55.66%)	Rank	PC2 (20.87%)	Rank	PC3 (18.74%)	Rank	Synthesis score	Rank
CM3	2.8117	1	-0.9369	3	-0.1418	2	1.4090	1
CM2	0.0265	2	1.6137	1	-0.7373	4	0.2237	2
CM1	-0.4217	3	0.4808	2	1.5162	1	0.1577	3
WM4	-2.4166	4	-1.1576	4	-0.6371	3	-1.7903	4

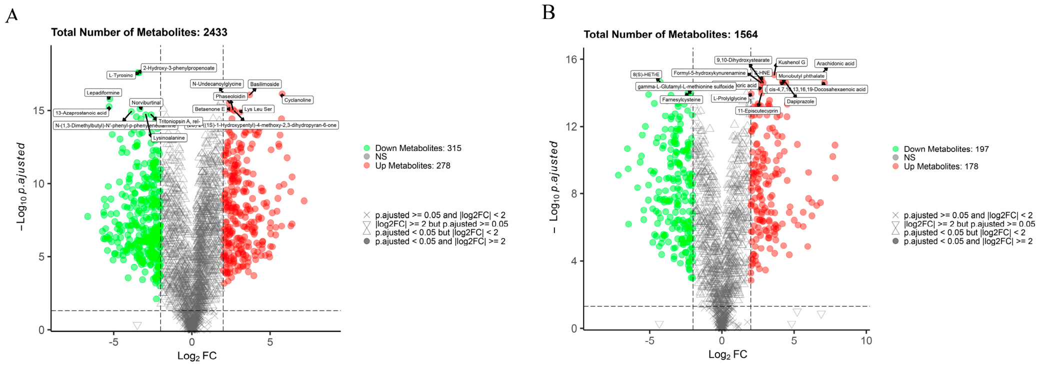

Figure S1. Volcano plot analysis of metabolites with differential abundance between *Agaricus bisporus* cultivated on corn stover-based substrate (CM3) and wheat straw-based substrate (WM4). (A): ESI+ mode; (B): ESI− mode.
